# Calmodulin in *Paramecium*: Focus on Genomic Data

**DOI:** 10.3390/microorganisms10101915

**Published:** 2022-09-27

**Authors:** Eduardo Villalobo, Gabriel Gutiérrez, Antonio Villalobo

**Affiliations:** 1 Facultad de Biología, Departamento de Microbiología, Universidad de Sevilla, Avenida Reina Mercedes 6, E-41012 Sevilla, Spain; 2 Facultad de Biología, Departamento de Genética, Universidad de Sevilla, Avenida Reina Mercedes 6, E-41012 Sevilla, Spain; 3 Cancer and Human Molecular Genetics Area—Oto-Neurosurgery Research Group, University Hospital La Paz Research Institute (IdiPAZ), Paseo de la Castellana 261, E-28046 Madrid, Spain

**Keywords:** calcium, calmodulin, cell signaling, ciliate, *Paramecium*

## Abstract

Calcium (Ca^2+^) is a universal second messenger that plays a key role in cellular signaling. However, Ca^2+^ signals are transduced with the help of Ca^2+^-binding proteins, which serve as sensors, transducers, and elicitors. Among the collection of these Ca^2+^-binding proteins, calmodulin (CaM) emerged as the prototypical model in eukaryotic cells. This is a small protein that binds four Ca^2+^ ions and whose functions are multiple, controlling many essential aspects of cell physiology. CaM is universally distributed in eukaryotes, from multicellular organisms, such as human and land plants, to unicellular microorganisms, such as yeasts and ciliates. Here, we review most of the information gathered on CaM in *Paramecium*, a group of ciliates. We condense the information here by mentioning that mature *Paramecium* CaM is a 148 amino acid-long protein codified by a single gene, as in other eukaryotic microorganisms. In these ciliates, the protein is notoriously localized and regulates cilia function and can stimulate the activity of some enzymes. When *Paramecium* CaM is mutated, cells show flawed locomotion and/or exocytosis. We further widen this and additional information in the text, focusing on genomic data.

## 1. Introduction

Cells adapt to the environment, which constantly changes, by means of chemical signals that act as messengers and whose concentration vary in time and space. In this sense, the calcium ion (Ca^2+^) emerged as a key, universal, versatile, and dynamic signal that regulates many cell functions [1]. The signal due to this ion, coming either from internal stores or from an external medium, switches on and off by, respectively, increasing and decreasing its concentration in the cytoplasm.

However, in most cases the corresponding signaling is not mediated by Ca^2+^ itself, but rather by different Ca^2+^-binding proteins [2]. The intracellular Ca^2+^ concentration, the result of the precise balance between the on and off mechanisms, is also due to the concerted action of a set of proteins, known as the Ca^2+^-signaling toolkit, constituted by buffers, pumps, channels, exchangers, receptors, transducers, sensors, and effectors [3]. The proteins of this set combine in different ways, crafting each signaling pathway and transmitting the Ca^2+^ signal temporally and spatially.

Calmodulin (hereafter abbreviated CaM) is considered an archetypal Ca^2+^ sensor, effector, and adaptor protein in eukaryotes [4], due to its ubiquity, conservation across species, and versatility, which ultimately allows it to target a huge number of proteins that in turn control many cellular functions [5]. By far, no other protein like CaM exemplifies the importance of Ca^2+^ in the evolution of signaling pathways in eukaryotes.

This article aims at reviewing CaM in the ciliate genus *Paramecium*, which are single-celled eukaryotes that have served for more than 100 years as model organisms for studying cilia, cytoskeleton, non-mendelian inheritance, genetic code, whole-genome duplications, epigenetics, endosymbiosis, and Ca^2+^ signaling, among other research topics. However, first, we will describe some general concepts about CaM and *Paramecium*.

## 2. Calmodulin

The name calmodulin stems from CALcium-MODULated proteIN, according to the educational portal of the Protein Data Bank (https://pdb101.rcsb.org/motm/44, accessed on 1 July 2022). CaM was first described, independently, by Cheung [6] and Kakiuchi and co-workers [7], as an activator of cyclic 3′,5′-nucleotide phosphodiesterase. However, the name was coined later by Cheung [8] once the relation between CaM and Ca^2+^ was clearly established [9].

CaM is a sensor for Ca^2+^, which is a key element in cell signaling. Indeed, CaM can bind up to four Ca^2+^ at the so-called EF-hand motifs (see Section 2.1). Thus, CaM can be seen as a transducer between Ca^2+^ signaling and the proteins that are the ultimate targets of the action of this divalent cation. Nonetheless, CaM can also bind and regulate a few targets in its Ca^2+^-free form (apo-CaM). Upon calcium binding, conformational changes in CaM entail the exposure in the surface of the protein of a series of hydrophobic patches, containing methionine residues [10], leading to the interaction with the CaM-binding proteins.

CaM is involved in the regulation of multiple signaling pathways that in turn control a variety of cellular functions, including metabolic control, cell proliferation, cell survival, differentiation, apoptosis, autophagy, and cell motility, among others [5]. Thus, it is not an overstatement to say that CaM binds hundreds of targets; among them are enzymes, receptors, ion channels, transcription factors, signaling proteins, adaptors, and structural proteins. In humans, for instance, CaM can bind at least 269 targets [11]. The target proteins have CaM-binding domains that show high sequence variability. In spite of this, most of these sequences are characterized by basic amino acid residues interspersed within bulky hydrophobic residues and bracketed by aromatic residues [3].

Having changed only slightly over 1.5 billion years of evolution [3], CaM is found in all eukaryotes, from humans to fungi, and from land plants to protists [8]. In all so-far-analyzed eukaryotes, mature CaM, lacking the starting Met, is 148 amino acid residues long. CaM is rich in acidic amino acids (Asp and Glu), and devoid of Trp and Cys [12]. CaM is a 16,790 Da thermostable protein with an isoelectric point between 3.9 and 4.3 [12].

The tertiary amino acid sequence of CaM folds in two globular lobes, located, respectively, at the N- and C-terminus, which are connected by a flexible linker. Two Ca^2+^ ions bind to each lobe at their EF-hand motifs, but the lobes can act independently from each other and interact with identical or different target proteins. This protein–protein interaction leads to homo-dimerization, or to the formation of new functional domains, if it occurs through different regions of the same protein [13]. The CaM-mediated interaction between different proteins usually leads to hetero-dimerization.

### 2.1. The EF-Hand Motif

The EF-hand motif was first described by Kretsinger and Nockolds in parvalbumin [14]. It is mostly 30 amino acids long, arranged into two perpendicularly alpha helices interrupted by a small 12 amino acids loop (helix–loop–helix), mimicking a right human hand with a spread thumb and forefinger, representing the E and F helices, respectively. Ca^2+^ is bound in a pentagonal bipyramidal geometry to the coordinating ligands, which are specific amino acid residues lying on the loop [15]. EF-hand proteins deal with all aspects related to cellular Ca^2+^, including signaling and homeostasis.

The EF-hand motif is distinctive of eukaryotes, though proteins with EF-hand-like motifs are also found in prokaryotes [16,17,18] and viruses [19,20]. The simplest EF-hand protein harbors two EF-hand motifs, connected by a linker, that form a four-helix package. In this manner, stability in the coordination is improved, leading to an increase in binding affinity towards Ca^2+^. From an evolutionary point of view, a paired motif is thought to have arisen by duplication of a single ancestral EF-hand motif. Indeed, in proteins with more than two EF-hand pairs, the highest amino acid similarities are found between alternate EF-hand motifs, namely, I–III and II–IV in the case of proteins with two EF-hand pairs. By far, EF-hand proteins are the largest family of Ca^2+^-binding proteins in eukaryotes. For instance, to date, up to 250 EF-hand proteins have been identified in humans, among them parvalbumin, myosin light chains, troponin C, and CaM.

A simple search for EF-hand proteins in the *Paramecium* database (ParameciumDB, https://paramecium.i2bc.paris-saclay.fr, accessed on 1 July 2022) returned 5924 entries and 13 protein domains; among them are the EF-hand domain pair PF13499 and PF13833 in Pfam and IPR011992 in INTERPRO. These databases are accessible at http://pfam.xfam.org and https://www.ebi.ac.uk/interpro/, respectively (accessed on 1 July 2022).

This review focuses on CaM; therefore, no additional information on the EF-hand motif-containing proteins will be included herein. The reader can consult the following general reviews for further information on the EF-hand [17,18,21,22,23,24,25,26,27,28].

## 3. *Paramecium*

Paramecia are ciliated, ovoid, elongated, free-swimming, single cells sometimes visible to the naked eye. These ciliates, like many others, have two structurally and functionally different nuclei, called the micronucleus and macronucleus. The micronucleus is diploid; it is the germline nucleus that transmits the genetic information to the offspring. The macronucleus is polyploid, and stems from the micronucleus after each sexual generation; it oversees the genetic expression, which determines the cell phenotype [29].

Like all ciliates, the genus *Paramecium* belongs to the Phylum Ciliophora [30]. Its species are further classified within Class Oligohymenophorea, Order Peniculida. Close relatives to the ciliates are, among others, the Aplicomplexa, such as *Plasmodium falciparum* (responsible for malaria), the Dinoflagellata, such as *Alexandrium tamarense* (responsible for some red tides), and Perkinsozoa, such as *Perkinsus marinus* (responsible for dermo disease in oysters).

The species concept in *Paramecium* challenges traditional species concepts, since morphology, mating behavior, and genetics features are discordant and not correlated in the lineage [31]. However, the discussion of this concept is beyond the scope of this article. Thus, in the easiest possible way, i.e., the morphospecies, the species of *Paramecium* fall in two groups according to their shape [32]: members of the “Aurelia” complex are characterized by a relatively long, cigar-shaped body, while those of the “Bursaria” complex are characterized by a shorter, broader, foot-shaped body. Furthermore, members of each group can be further differentiated by the shape of their micronuclei: the “Caudatum” type has a large and ellipsoidal micronucleus, whereas the “Aurelia” type has a spheroid, small, vesicular micronucleus [32]. According to taxonomy of the cell shape and nuclear characteristics, at least 15 species can be identified in *Paramecium*.

The macronuclear genome sequences of several *Paramecium* species have been obtained; among them are *P. tetraurelia* (the first to be obtained in 2006 [33]), *P. biaurelia*, *P. bursaria*, *P. caudatum*, *P. decaurelia*, *P. dodecaurelia*, *P. jenningsi*, *P. novaurelia*, *P. octaurelia*, *P. pentaurelia*, *P. primaurelia*, *P. quadecaurelia*, *P. sexaurelia*, and *P. sonneborni*. Macronuclear genome sequences for the different stocks of some of these species also have been made available. In addition, the micronuclear and mitochondrial genome sequences are also available for a few of these species. All data are publicly and freely available at ParameciumDB (see also [34]). Likewise, the macronuclear genome sequences of some other ciliates have been obtained and are freely searchable.

**Figure 1:**
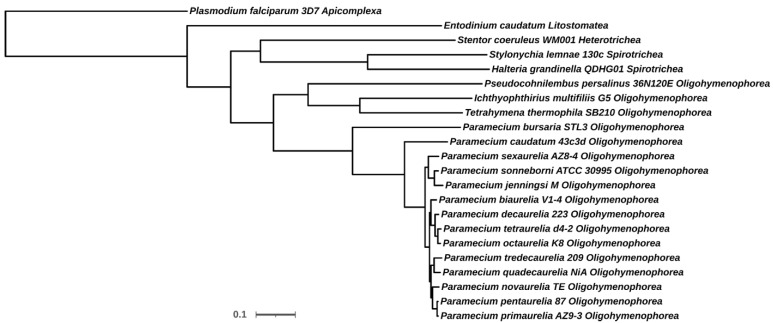
Phylogenetic tree of selected species of ciliates. Protein datasets were downloaded either from ParameciumDB or NCBI, except for *Entodinium caudatum*, which was kindly provided by Drs. Yu and Park from Ohio State University, OH, USA. All proteomes were used for feeding Orthofinder (see text above for reference). A total of 507 orthogroups with all the species was used to build the tree, using the STAG algorithm included in Orthofinder. The inferred tree was drawn with iTOOL [36]. *P. falciparum*, an apicomplexan close relative of ciliates, was selected as outgroup by the STRIDE algorithm, also included in Orthofinder. For each species, the full name, strain, and Class to which each species belongs are shown, except for *P. falciparum*, for which the Phylum was written instead of the Class. The scale bar represents the average number of substitutions per sites across of the 507 orthogroups used.

Harnessing the huge amount of genomic data available on *Paramecium*, we decided to re-examine the phylogenetic relationship among the species in *Paramecium*. To do so, we built a phylogenomic protein tree (Figure 1) using the Orthofinder tool [35]. This tool groups the annotated proteomes in the so-called orthogroups and builds a consensus tree based on them. According to Orthofinder, the orthogroups are the set of genes descending from a single gene in the last common ancestor of all the species in the analysis. However, by definition, the analysis includes both orthologs and paralogs. In our analysis, a total of 41,662 orthogroups were raised, one of which contained CaM sequences (see Section 4.5 and Section 4.8).

Overall, the clustering observed in the tree (Figure 1) is coincident with the clustering that have been published previously using single-protein analysis [37]. This clustering can be described as a bush from which Class Oligohymenophorea emerged as a monophyletic lineage. This monophyletic pattern is congruent with what has been published using rRNA sequences [38,39]. In the tree, Class Oligohymenophorea, at the same time, appeared subdivided into three different groups, corresponding to Order Peniculida (*Paramecium*), Hymenostomatia (*Tetrahymena thermophila* and *Ichthyophthirius multifiliis*), and Scuticociliatia (*Pseudocohnilembus persalinus*). As expected, all *Paramecium* species grouped together, as a single clade, in which the “Aurelia” and “Bursaria” complexes formed two different subgroups of species. In addition, within the “Aurelia” complex, *P. caudatum* appeared separated from the other species. This separation has also been observed by others using single-protein analysis [40].

### 3.1. Motility and Ion Channels

Paramecia are single-celled eukaryotes that move and feed thanks to cilia, which decorate the cell surface. The movement of this ciliate is described as a “swim”, in contrast to the “crawl” observed in other ciliates, such as *Euplotes*. Paramecia usually and persistently swim forward. The forward swim occurs because cilia beat differently following the anterior–posterior axis; cilia strike stronger toward the posterior and idly toward the anterior axis when coming back [41,42]. However, to move away from negative stimuli, cells tend to shortly swim backwards, then twirling to change the direction, and swimming forward again; cells repeat this pattern until the negative stimuli are left out. This latter behavior has been called the “avoiding reaction” [43]. During backward swimming, cilia beating reverses with respect to forward swimming; i.e., a stronger stroke is towards the anterior, while the idler stroke is towards the posterior axis [41,42].

Ion conductance and potential through the cell membrane control the frequency, speed, and direction of a ciliary stroke. Frequency and speed of a ciliary stroke during normal forward swimming depends on the resting membrane potential. Those stimuli that hyperpolarize the cell slightly increase the ciliary stroke toward the posterior; consequently, the cell moves forward faster. Conversely, stimuli that depolarize the cell have the opposite effect, reversing the direction of the ciliary stroke and, hence, the cell moves backward.

Molecularly, the depolarization above the threshold causes an increase of intraciliary Ca^2+^ above 100 nM, due to the opening of voltage-gated Ca^2+^ channels (Ca_v_) located exclusively in the ciliary membrane. Then, a rapidly activated voltage-gated K^+^ conductance (I_Kv_) is concentrated in the cilia. Finally, a slower Ca^2+^-activated K^+^ conductance (I_KCa_) acts to return the membrane potential to the resting level via K_Ca_ channels, with the Ca^2+^ coming from the Ca_v_ channels of the cilia [44]. The backward swim lasts as far as the Ca^2+^ return to low nM levels, which is accomplished, at least in part, by plasma membrane Ca^2+^-ATPase pumps [45].

*Paramecium* has at least eight kinds of ion channels [46], with different triggering mechanisms, on–off-kinetics, and ion selectivity, some of which are located in the plasma membrane and others in the ciliary membrane [44,47,48]. There are three voltage-gated Ca^2+^ channels [49], and two channels regulated by Ca^2+^, one passing Na^+^ inwards and one passing K^+^ outwards.

The locomotion of *Paramecium* serves as a distinctive trait for normal ciliary function and swimming behavior (behavioral response). Therefore, cells affected in locomotion are readily detectable by observing their behavior under a microscope. Along the years, collections of behavioral mutants were obtained and analyzed [50]. Electrophysiology revealed that most of these mutants had a faulty membrane function (reviewed in [50]). The first group of mutants (called “paranoids”) were characterized by an exaggerated reaction to Na^+^ and episodes of prolonged and violent backward swimming. The second group of mutants (called “Fast-2”) lacked backward swimming in the presence of depolarizing Na^+^ solutions. In both mutant types, the Na^+^ conductance is affected due to an uneven Ca^2+^-activated Na^+^ current. The third group of mutants (called “pantophobiac”) had excessive responses to mild depolarizing solutions, having episodes of minute-long backward swimming. In these mutants, the lack of a K^+^ current, activated by Ca^2+^, delayed the repolarization of the cell to the normal resting potential.

The overall importance of the Ca^2+^ ion, and its corresponding channels, in the swimming behavior of *Paramecium* is beyond all doubts. Thus, it was only a matter of time that research on mutants affected in swimming behavior led to the implication of CaM in locomotion, as many ion channels are regulated by CaM.

### 3.2. The Cortex and the Secretion Systems: The Trichocyst

The cortex [51] is the semi-rigid external layer of the cells, functioning like a flexible skeleton that can bend and return back to its initial position to permit cell body movements. It is a semi-permeable layer that allows substances to flow through and that senses mechanical, chemical, and electrical stimuli from the surroundings. The cortex is made of several structures that are, from the outside to the inside, the plasma membrane, which is continuous over the cilia; the alveolar system, which are flattened and membrane-bounded sacs constituting calcium stores [52]; and the epiplasm, which is an amorphous and granular layer lining the alveolar system. These three layers outline the ridges and furrows that form a pattern of hexagons and parallelograms. The sides of the ridges are linked and stabilized with short bundles of microfilaments, called the striated band, that insert into the epiplasm.

The cilia, singly or in pairs, emerge from the furrow made by the ridges, and are arranged in rows throughout the cortex. A cilium can be considered an outgrowth of the basal body, which is the homologous structure to the centriole in animal cells. Internal to the striated band, at the level of the basal bodies, is located the infraciliary lattice, bundles of branched fine filaments that do not associate with any membrane.

Trichocysts (for reviews, see [53,54]), the main secretory organelles of the cell, are docked within ridges beneath the plasma membrane. They are needle-shaped structures when extruded, but carrot-shaped when docked, with the tip surrounded by a sheath. The trichocyst body is made of a mass of proteins, called the trichocyst matrix proteins. A fibrous material, anchored through a rosette of intramembranous particles, connects the plasma and trichocyst membrane.

Upon external stimuli, trichocysts eventually fuse to the plasma membrane, extruding their contents towards the exterior. A collection of mutants helps to disentangle biogenesis, docking, and release of trichocysts. These mutants have been classified according to their main defect; for instance, those showing non-discharge were named nd, while those having no visible trichocysts, trichless mutants, were named tl. Alternatively, some behavioral response mutants were shown to be defective in secretion (see Section 4.5.1 and [55]).

Apparently, resting trichocysts have no free Ca^2+^ [54], though this ion has a main role in their extrusion (see, for instance, [53,54,56,57]). Indeed, it has been proposed that trichocyst release might require more than 1 μM of Ca^2+^ [58]. Moreover, a CaM-dependent ATPase [59,60], among other Ca^2+^-related proteins, was found to be implicated in trichocyst extrusion. Thus, it is not surprising that the search for CaM in trichocysts was encouraged.

## 4. Calmodulin in *Paramecium*

Largely, we compiled the information provided by two resources: the NCBI databases (https://www.ncbi.nlm.nih.gov, accessed on 1 July 2022) and the genomic database for different *Paramecium* species, here referred to as ParameciumDB (https://paramecium.i2bc.paris-saclay.fr, accessed on 1 July 2022). A restrictive search at NCBI for “paramecium [Title/Abstract] AND calmodulin [Title/Abstract]” returned 101 citations in the PubMed database. After mining the results, we found that the earliest evidence of CaM in *Paramecium* dated back to 1979–1980 (the first evidence appeared as a conference abstract in Maihle and Satir 1979 [61]; see also [62]). In addition, we found that not all citations specifically dealt with *Paramecium* or CaM. We found many reviews, most of them addressing CaM marginally. From all these reviews, only three explicitly deal with CaM [63,64,65], of which two have to do with *Paramecium* expressly [64,65].

A simple search for “calmodulin” or “CaM” in the ParameciumDB gave too many entries to be shown. Thus, we gathered the information from this database using some other tools, such as Blast. Below, we summarize most of the information unearthed from both the bibliographic and genomic resources. In order to facilitate the reading, we have organized and grouped the information on several topics.

### 4.1. Cellular Localization

The information on cellular localization of CaM in *Paramecium* is dispersed, since we have found at least five papers on this issue. In the first report [62], localization of CaM was carried out by means of an anti-CaM antibody raised against rat CaM obtained from testes [66]. However, in the first report, no images were given to support the author’s claim, stating that not only *Paramecium* CaM but also that of *Tetrahyemena* were localized in the cilia and cortex [62]. In the second report [67], which dealt with *Paramecium* only, images showed strong labelling in Ca^2+^-containing birefringent vesicles (perhaps food vacuoles), in oral and somatic cilia, and in linear, punctuate spots, corresponding to kineties in deciliated cells. The third report, in 1983 [68], confirmed the labelling patterns mentioned above.

The localization of CaM was reported once again in 1986 [69], but this time using polyclonal antibodies raised against purified CaM from *Paramecium*. In this report, the ultrastructural level was included. After injecting the antibodies into living cells, several structures were labelled, such as the cortex, the food vacuole, and some structures of the osmoregulatory system, including the contractile vacuole. However, fixing the cells after injecting the antibodies changed the labelling pattern, since the infraciliary lattice, the inner most structure of the cell cortex, seemed to be revealed. In thin sections, the fine filaments of the infraciliary lattice were again revealed by the antibodies, as well as the membrane of trichocysts, which are organelles found in the cortex and that can be extruded upon certain stimuli (see above Section 3.2). This latter observation agreed with the fact that CaM had been shown to be an integral part of extruded trichocysts [70]. Noteworthy, an initial report failed to stain trichocysts by CaM, though in that case the labelling was through FITC-marked CaM or trifluoperazine, which is an autofluorescent CaM-antagonist [71]. Further observations in ultrathin sections [69] supported the labelling of the abovementioned structures, including trichocysts and cilia. Previously, CaM had been successfully isolated (see below, Section 4.2) from purified cilia [72] and extruded trichocysts [70]. However, these findings should not be interpreted as unequivocally affirming that CaM is an integral part of the trichocysts (see the discussion in the following section).

Finally, in a different species of *Paramecium*, CaM was seen to be localized in practically all the structures we have mentioned in Section 4.1, though some previously un-labelled structures became evident, such us the cytopharyngeal microtubular ribbons, the postoral fiber, and the cytoproct microtubules [73]. In this latter report, an antibody raised by Burgess-Cassler and collaborators [74] was used in immunogold staining on cryosections. Similarly, immunofluorescence staining, using an antibody raised against *Tetrahymena* CaM, showed that the protein was localized into the oral apparatus, cilia, basal bodies, anterior end of the cell, and contractile vacuole pore [75].

### 4.2. Isolation and Purification

Similar to localization studies, biochemical studies have been profuse in *Paramecium*. Thus, many papers have reported about the isolation and/or purification of CaM in this ciliate. The methods used to obtain the protein were varied from simply heating cells at 80 °C and then precipitating with ammonium sulfate [62], through phenyl-Sepharose chromatography (see, for instance, [69]), right up to immunoaffinity chromatography (see, for instance, [74]). Sometimes, CaM was isolated and purified from subcellular structures, such as cilia [72] or extruded trichocysts [70]. Nonetheless, we would like to mention that it was reported elsewhere that the gradient-purified trichocyst matrix did not contain CaM, according to its detection by an anti-CaM antibody [76]. These authors argued that CaM was not a major component of the matrix of trichocysts, though its amount might be below the detection threshold of the anti-CaM antibody. Taken together, these results, and those shown in the preceding section, suggest that CaM is either weakly associated with trichocysts or not present at all, as claimed elsewhere [54].

Due to its importance in clarifying the role of CaM in *Paramecium*, we would like to mention in more detail a few papers about pantophobiac mutants, in which different CaM preparations were obtained, including a highly pure one. Pantophobiac mutants, cells affected by locomotion, are characterized by the loss of the Ca^2+^-dependent K^+^ current, which shuts off Ca^2+^ excitation specifically. Therefore, the loss of this current causes a prolonged membrane excitation, resulting in exaggerated locomotion responses to different stimuli, hence the name pantophobiac (these mutants are mentioned later in Section 4.5.1). Since the current was dependent on Ca^2+^, the issue on whether CaM was responsible for the mutant phenotype was pertinent and logic.

The first paper [77] showed that microinjection of the fractionated wild-type cytoplasm, consisting of a supernatant after high-speed centrifugation, in pantophobiac mutants turned their locomotion to normal. The recovery of the phenotype was concomitant with the restoration of the Ca^2+^-dependent K^+^ current, which was defective in the mutant. According to analysis by electrophoresis and Coomassie staining of supernatants, a visible protein band in the range of 17 kDa was observed after heating. This protein changed its mobility in SDS-PAGE in the presence of excess Ca^2+^ in the buffer. This Ca^2+^-dependent, electrophoretic shift is a property characteristic of CaM [78], and it has been observed in *Paramecium* (see, for instance, [67]). This band was absent when excess EGTA was added to the buffer. This is expected because CaM precipitates when heated in the presence of EGTA, a Ca^2+^-chelating chemical [79]. Reasonably, wild-type, heat-treated, EGTA-containing cytoplasm was unable to recover the locomotion defects of pantophobiac mutants.

In the second paper [77], CaM was successfully purified to electrophoretic homogeneity, not only from wild-type cells, but also from a pantophobiac mutant. The purification procedure included DEAE-cellulose anion exchange and Phenyl-Sepharose column chromatography, and gave rise to a single 16.5 kDa protein. The amount of cytoplasmic CaM in *Paramecium* was estimated elsewhere to be about 5 ng/μg protein [80] or 1.5% cellular protein [81]. The purified CaM from wild-type cytoplasm was able to restore the defects of the pantophobiac mutant, while the purified CaM from mutant cytoplasm did not, neither did CaMs from other organisms, such as bovine or *Dictyostelium*. Strikingly, both wild-type and pantophobiac CaM had comparable phosphodiesterase-stimulating activities (see below, Section 4.3), despite subtle differences being observed and accounted for later [74].

Overall, it was concluded that the phenotype of pantophobiac mutants did not necessarily relate to the CaM gene itself, because it might be a gene coding for either a CaM-modifying protein or a CaM-like protein, whose function was mimicked by CaM. Since these results were puzzling, soon they prompted the sequencing of CaM (see below, Section 4.5).

### 4.3. Enzyme Targets

CaM is able to stimulate the activity of numerous enzymes, mostly in its Ca^2+^-saturated form, although some systems are also regulated in its Ca^2+^-free form (see, for instance [13,82,83,84]). Among the enzymes, it activates 3’,5’-cyclic AMP phosphodiesterase (PDE), myosin light chain kinase (MLCK), guanylyl cyclase (GC), plasma membrane Ca^2+^-ATPase (PMCA), and calcineurin (CaN). The stimulation of PDE activity (mammalian enzyme) has been recognized since the earliest studies on CaM [6]. Even *Tetrahymena*, a close relative to *Paramecium*, was shown to possess CaM with this stimulating activity [85].

The fact that *Paramecium* CaM had the PDE-stimulating activity was independently demonstrated several times, either with cell homogenates or with purified proteins. Here, we will mention a few papers to exemplify how CaM stimulates the activity of this enzyme. One of the very first demonstrations [62] showed that heat-treated cell homogenates containing CaM were able to stimulate PDE activity. This activity in homogenates was clearly inhibited by trifluoperazine, a CaM antagonist [71]. This was remarkable, as many inhibitors for mammalian enzymes fail to inhibit enzymes in ciliates. A decade later, it was shown [86] that trifluoperazine was unable to inhibit the PDE-stimulating activity of purified CaM, although some other stronger CaM antagonists (e.g., W-7, a naphthalenesulfonyl derivative) did have inhibitory effects [86,87]. Furthermore, the naphthalenesulfonyl derivatives affected the behavioral response of cells, while trifluoperazine did not. This response depends on Ca^2+^ channels (see above, Section 3.1) that, in turn, are directly or indirectly controlled by CaM. However, trifluoperazine and W-7 did have inhibitory effects on trichocyst discharge [80,88]. As stated above in Section 4.1 and Section 4.2, CaM had been successfully purified [70] from and localized [69,70] in extruded trichocysts.

The idea that purified CaM had PDE-stimulating activity came true subsequently. To evidence this, two different purification procedures were used, either immunoaffinity purification [74] or the supernatant of a high-speed centrifugation followed by DEAE cellulose anion exchange and phenyl-Sepharose chromatography [77]. In the case of immunoaffinity-purified CaM [74], wild-type CaM and mutant CaM (pantA^1^, see below in Section 4.5.1) were compared to each other, resulting in slight differences in PDE activity. This difference consisted of that the mutant version did not stimulate PDE activity at low Ca^2+^ concentrations to the same extent than the wild type did. This report showed that mutant and wild-type versions also differentiated in two other biochemical properties. On the one hand, their mobility in acid-urea gel electrophoresis; the mutant CaM was shown to run faster than its wild-type counterpart [74]. On the other hand, in quantitative ELISA assays using antibodies raised against wild-type CaM; 4 to 5 times more amount of mutant CaM was needed to give a comparable signal to that of the wild-type CaM. This result, along with competition experiments with ^125^I-radiolabelled CaMs, led to the conclusion that the antibodies bound more effectively wild-type CaM than mutant CaM.

A Blast search in ParameciumDB against human PDE raised several proteins that could code for PDE in *P. tetraurelia* (stock d4-2). Among them, the protein annotated as GSPATG00036826001 contains the Pfam domain PF00233, which represents cyclic nucleotide phosphodiesterases. This protein has two paralogs derived from whole genome duplications (GSPATP00021356001 and GSPATP00032825001). This Pfam domain is present in at least 40 different protein entries in this stock of *P. tetraurelia*.

The stimulation of MLCK activity by CaM was also demonstrated in *Paramecium*, specifically for mutant CaM versions, since MCLK activity is sensitive to mutations in some surface-exposed, hydrophilic residues of CaM [89,90]. A SYNCAM version containing an Ile136Thr mutation, similar to a mutant *Paramecium* CaM (pantA^2^, see below Section 4.5.1), was able to stimulate MLCK activity to a level comparable to that of SYNCAM [91]. SYNCAM is a synthetically designed recombinant CaM that, compared to vertebrate CaM, possesses enhanced activity towards many CaM-regulated enzymes [90]. Similarly, it was shown that a SYNCAM version, mimicking a mutation Ser101Phe in *Paramecium* CaM, was able to stimulate MLCK activity to a level comparable to that of the non-mutated SYNCAM [92]. Further information on kinases can be found below in Section 4.3 (see also [93]).

A Blast search in ParameciumDB against human MLCK raised several proteins that could code for MLCK orthologs in *P. tetraurelia* (stock d4-2). Up to three of these entries are annotated as calcium-dependent protein kinase 1, while five other entries are annotated as containing a protein kinase domain. For instance, GSPATG00020375001 contains several Pfam domains related to kinases (PF00069, PF07714, PF14531, and PF01636) and has three paralogs from whole-genome duplications (GSPATG00023613001, GSPATG00010951001, and GSPATG00014433001).

A Ca^2+^-sensitive GC found in the ciliary membrane of *Paramecium* [94] was associated to CaM [95]. This GC was partially inhibited (30%) by trifluoperazine [95]. The weak inhibitory effect of this compound on GC, in comparison to other CaM-stimulated enzymes, was explained by the supposed differences between *Paramecium* and mammalian CaMs. Indeed, it was also shown [95] that purified *Paramecium* CaM needed 5-fold more trifluoperazine than pig CaM to inhibit PDE activity. As little as 0.5 μg CaM was enough to obtain half maximal activation of lanthanide-treated GC. The rationale for this assay was the assumption that CaM is tightly bound to the GC and, thus, addition of lanthanide should entail the removal of CaM from GC preparations, giving CaM-free GC to perform dose-dependent assays [95]. Similarly, these authors found [96] that lanthanide-treated, *Paramecium* GC was dose-dependent activated by several CaMs, including brain CaM and a genetically engineered CaM, lacking trimethyl-lysine at residue 115. Strikingly, when GC devoid of CaM was reconstituted, trimethyl-lysine-lacking CaMs failed to restore the enzyme activity [97]. This result should be interpreted carefully, since the reconstituted GC had some other remarkable differences; for instance, Ca^2+^ was not the best suited ion for enhancing CaM-dependent activity, since Sr^2+^ was the most effective ion in doing so [97].

Eukaryotes possess two different GC, a soluble and a membrane-bound type (for a review, see [98]). As mentioned above, the activity of GC was detected in *Paramecium* (for a review, see [99]), and the nucleotide sequences of two-domain integral membrane GC were obtained [100]. We retrieved, from the genomic database, a membrane-bound GC in *P. tetraurelia* (annotated as GSPATP00017090001) and noticed that it had another paralog from the whole-genome duplications (annotated as GSPATP00035245001). However, this sequence was not annotated as a GC and was longer (2417 amino acids) than its counterpart (2267 amino acids). An alignment of these two protein sequences gave a 93.79% similarity along 2417 amino acids (alignment not shown). To ascertain if *Paramecium* has a soluble GC, we inquired the genomic database again, and found no clear evidence of such an enzyme type, but rather we found many other sequences annotated as adenylyl cyclase class-3/4/guanylyl cyclase, though the similarities with GSPATP00017090001 ranged from ca. 52%, to 44% after pairwise alignments (alignment not shown).

PMCA is another enzyme regulated by CaM, which interacts with high affinity to the cytosolic C-terminal tail of PMCA and acts as an activator [101]. Apparently, CaM also binds with lower affinity to a second domain, located downstream of the first one. A PMCA was successfully isolated from membrane pellicles from *Paramecium* [102], and its corresponding gene obtained [103]. The deduced amino acid sequence of *Paramecium* PMCA showed the canonical CaM-binding site sequence, besides some other recognizable sites, such as the ATP binding site, the acylphosphate formation site, and the so-called hinge region [103]. The corresponding purified enzyme was specific for ATP as substrate, required Mg^2+^ for optimal Ca^2+^ stimulation, and its Ca^2+^-stimulated activity was inhibited by vanadate, while sodium azide, oligomycin, or ouabain did not, as expected. Furthermore, calmidazolium and trifluoperazine also acted as inhibitors. Despite these two latter chemicals are well-known antagonists of CaM [71,104], it was noted that bovine CaM did not stimulate the activity of *Paramecium* PMCA. *Paramecium* also possess a Ca^2+^-sensitive Mg^2+^-ATPase in their cilia, which was shown to be insensitive to CaM antagonists [105]. While it seemed that CaM was present in axonemes, where this ATPase activity was detected, CaM was not associated with ciliary dynein fractions. Probably, CaM might bind some axonemal structures, possibly related to a different ATP-hydrolyzing activity, though this was not further verified.

Many protein kinases are dependent on Ca^2+^ for their activities, among them Ca^2+^/calmodulin-dependent protein kinases are of special interest, because they require Ca^2+^-bound CaM for its activation [106]. *Paramecium* has two of these kinase activities, which have been purified and characterized [107,108]. The activity of one of these kinases [107], named Ca^2+^-dependent protein kinase-2, was completely dependent on Ca^2+^, but not on phosphatidylserine and diacylglycerol. Some of its targets were casein and histones, while it also autophosphorylated. After denaturation/renaturation experiments, the enzyme retained its ability to phosphorylate casein in a Ca^2+^-dependent manner, which implied Ca^2+^ should interact with the protein directly. In fact, *Paramecium* CaM did not stimulate its kinase activity. However, subsequently, CaM was shown to be phosphorylated in vitro by this Ca^2+^-dependent protein kinase-2 [109], though melittin and calmidazolium prevented CaM phosphorylation. CaM contains five possible Ser residue targets among its 148 amino acids at positions 38, 70, 81, 101, and 147, but results suggested that only residue 147 was phosphorylated. Finally, it was shown that phosphorylation did not prevent CaM from binding Ca^2+^.

Likewise, *Paramecium* CaM had no stimulatory effect on Ca^2+^-dependent protein kinase-1 activity; moreover, high concentrations of CaM had a slightly inhibitory effect [108]. Despite Ca^2+^-dependent protein kinase-1 and Ca^2+^-dependent protein kinase-2 shared many properties, they seemed to be different in some other properties. Some other kinases were also isolated from *Paramecium*, among them a Ca^2+^-inhibitable casein kinase, which was unable to phosphorylate *Paramecium* CaM [110].

To date, *P. tetraurelia* is the eukaryote with more genes dedicated to codifying protein kinases, a total of 2602, representing 6.6% of its genes [93]. Strikingly, among the 40 domain architectures that *P. tetraurelia* kinases use, the Ca^2+^-CaM kinase subfamily has massively expanded, indicating the importance of Ca^2+^ in this ciliate. How many of these kinases are indeed regulated by CaM is currently unknown.

As with many protein kinases (see earlier in this section), some protein phosphatases are also regulated by CaM; CaN [111] is perhaps one of the best studied of these phosphatases. CaN is a serine/threonine phosphatase that binds not only CaM but also Ca^2+^. Indeed, CaN is only partially active at a certain Ca^2+^ concentration and becomes fully active upon CaM binding. CaN is a heterodimer, constituted by chain A, containing the catalytic domain and the CaM-binding domain, and the regulatory chain B, containing the Ca^2+^-binding domain. Chain A is a polypeptide of about a 58–64 kDa, while chain B is around 19 kDa in size. Interestingly, chain B has four EF-hand motifs that allow the binding of four Ca^2+^ ions. Different heterodimers of CaN can be assembled because chain A has three different isoforms, named α, β, and γ.

It was first evidenced by Western blot [81] and thereafter by immunogold localization [112] that *Paramecium* has CaN. Subsequently, the genes corresponding to chains A and B were identified [113]. As reported, 7 gene subfamilies are responsible for codifying chain A, each subfamily containing 2 paralogs; i.e., 14 different genes codify for the catalytic subunit. Conversely, only two paralogs codify for the regulatory subunit. Information on genes coding for chain A are found in ParameciumDB as CaNA, under the following accession numbers (*P. tetraurelia* stock d4-2): GSPATG00015557001, GSPATG00012128001, GSPATG00017910001, GSPATP00022161001, GSPATP00019553001, GSPATP00010670001, GSPATP00032583001, GSPATP00033854001, GSPATP00022245001, GSPATP00020009001, GSPATP00002809001, GSPATP00001220001, GSPATP00021591001, and GSPATP00023268001 (respectively for CaNA1a, CaNA1b, CaNA2a, CaNA2b, CaNA3a, CaNA3b, CaNA4a, CaNA4b, CaNA5a, CaNA5b, CaNA6a, CaNA6b, CaNA7a, and CaNA7b). Similarly, the genes for the chain B are found in ParameciumDB as CaNB, under the accession numbers GSPATP00006336001 and GSPATP00009660001 (respectively for CaNB1a and CaNB1b).

By means of RNA interference, it was shown that silencing the CaNA1 genes, but not those of CaNA3, avoids trichocyst discharge in 25% of wild-type, RNAi-treated cells. The implication of CaN in trichocyst discharge had previously been suggested using an anti-CaN antibody [81], which inhibited discharge in isolated cortices. The effect of CaNA1 gene silencing was correlated with an increase in the time necessary to decay the Ca^2+^ concentration after stimulation of exocytosis [113]. In addition, it was shown that silencing the CaNA3 genes, but not those of CaNA1, increased backward swimming after applying a stimulus to wild-type, RNAi-treated cells. Taking these results together, it was concluded that the functions of the different CaN genes were not redundant and, therefore, each CaN isoform could target a specific set of substrates controlling specific cellular functions.

### 4.4. Other Protein Targets

Besides the abovementioned enzymes, CaM has a huge number of other proteins under its control. In its Ca^2+^-bound form, more than 250 different targets are known in humans [114], including ion channels, G-protein-coupled receptors, and many others implicated in diverse signaling pathways. In this way, CaM takes part in the regulation of many cellular processes, including metabolism, motility, proliferation, and differentiation, among many others.

As mentioned above (see Section 3.1), *Paramecium* possesses two Ca^2+^-regulated channels, one passing Na^+^ inwards and one passing K^+^ outwards [46]. The activity of single Ca^2+^-dependent Na^+^ channels was measured in excised patches of the plasma membrane from *Paramecium* [115]. It was shown that the activity of this channel decreased upon short-term exposure to low Ca^2+^ concentrations. Interestingly, the activity of the channel was practically recovered by adding purified CaM to the surface of the patches. However, the activity of the channel upon CaM recovering was lower than the activity of the channel prior to short-term Ca^2+^ exposure. The addition of bovine CaM had almost the same effect on channel reactivation. This reactivation was independent of ATP addition, indicating that the reactivating activity was not due to a Ca^2+^/CaM-dependent protein kinase. Overall, the authors have argued that Ca^2+^-CaM can directly activate the channel, though they did not exclude that CaM was bound to a different membrane protein that, in turn, activate the channel. To our knowledge, no direct evidence in *Paramecium* of the binding of CaM to this channel, or another target, has been gained yet.

Myristoylated, alanine-rich C kinase substrate (MARCKS) protein is also a target of CaM [116,117,118]. MARCKS binds actin and its function is regulated by protein kinase C (PKC), both in vivo and in vitro [116,117,118]. MARCKS cycles between the membrane and the cytosol, and it has been implicated in cell motility, secretion, membrane trafficking, and mitogenesis [116,117,118].

The primary structure of MARCKS can be subdivided into three different regions of conserved sequences [117,118]: the first region at the N-terminus, which is where the protein is myristoylated; the second internal region; and the third region, which contains the sites of phosphorylation by PKC. This latter region, also called the phosphorylation site domain (PSD), is made of 25 amino acids, most of which are of basic nature. Four amino acid residues are the potential targets by PKC, though only the first, second, and fourth Ser residues are indeed phosphorylated. Interestingly, the PSD is also a CaM-biding site, although CaM, in its Ca^2+^-bound state, can only bind PSD when it is not phosphorylated.

In the framework of *Paramecium* motility behavioral studies, there was an attempt to establish a relationship between CaM and PCK through MARCKS [119]. To do this, MARCKS-derived peptides were injected into wild-type cells and their behavioral response analyzed. It was first observed that a MARCKS peptide (KKKKKRFSFKKSFKLSGFSFKKNKK) was phosphorylated in a Ca^2+^- and phospholipid-dependent manner by a nonidentified *Paramecium* kinase, which was present in a cytosolic, unpurified cell extract (though this datum was not shown in the paper). When this MARCKS peptide was injected into cells, the normal duration of backward swimming increased after depolarizing stimuli were applied. Moreover, a transient and similar effect was seen upon injection of the intact human MARCKS protein, though again data were not shown. The slight difference was endorsed to proteolysis of the intact protein in the cytoplasm of the paramecia. Injection of MARCKS peptide in the presence of phorbol 12-myristate 13-acetate (PMA), a known activator of PCK, caused a modest increase in the duration of backward swimming. This latter was considered as indicative of the disruption by PKC of a putative peptide-calmodulin complex. To further prove this claim, a mutated MARCKS peptide (all the four Ser were changed to Ala) was injected into cells. Apparently, this tetra-Ala peptide was able to bind CaM but was not phosphorylated by PKC (unpublished datum by collaborators of the authors of this paper). After injection, it was observed that both the tetra-Ser and the tetra-Ala peptides had indistinguishable effects. However, addition of PMA had the same effect than its absence, while injecting the tetra-Ala peptide. As expected, the injection of a tetra-Asp peptide (mimicking the phosphorylated peptide) had no effect on the swimming behavior and had a 10-fold lower CaM-binding affinity than its tetra-Ser counterpart. Finally, co-injection of CaM prevented the increase in backward swimming duration that should have caused the tetra-Ser peptide.

We decided to search for MARCKS protein orthologs in *Paramecium*. To do that, on the one hand, we blasted against the *P. tetraurelia* protein database, the sequence of the CaM-binding/phosphorylation domain (KRFSFKKSFKLSGFSFKK), but no result was obtained. On the other hand, we retrieved the human MARCKS protein sequence and, again, after Blast search in *Paramecium*, no result was obtained. Therefore, if PKC acts jointly with CaM to regulate locomotion in *Paramecium* should be further assessed.

The presence of other CaM-binding proteins was reported, but their nature remains to be determined. The first set of CaM-binding proteins [120] were present and enriched in ciliary fractions—some of them further enriched after ciliary sub-fractionation. These proteins were classified in two types according to the range of Ca^2+^ concentrations at which they bind CaM. The first group bound CaM above 0.5–1 μM, while the second group did so below 1–2 μM. The proteins were named after their apparent sizes in SDS-denaturing gel electrophoresis, which were 126, 105, 95, 96, 63, and 36 kDa. Fractionation experiments showed that C126, E105, E95, C96, and C63 were axoneme-bound proteins, whereas C36 a soluble and/or membrane-associated protein. Note that C proteins are those that bound CaM in the submicromolar range, while E proteins did it in the micromolar range.

Another CaM-binding protein, called PCM1, was also obtained from ciliary membranes [121]. This protein was obtained after passing membranellar protein extracts through CaM–Sepharose in the presence of Ca^2+^ and eluting with EGTA; after elution, PCM1 was the only detectable protein in gel electrophoresis, showing a size of about 65 kDa. Blot overlay experiments, using ^35^S-CaM and a high Ca^2+^ concentration, further demonstrated that PCM1 binds CaM in its high affinity form. It is well known that CaM can bind target proteins either in its Ca^2+^-free, half-saturated, and fully saturated forms [122]. PCM1 was purified from ciliary membrane vesicles, but not from cytosolic fractions. Sequences from protease-digested peptides of PCM1 were obtained, which allowed the corresponding gene to be determined. During the identification of the PCM1 gene (annotated as GSPATG00029566001 in the protein database of *P. tetraurelia*), three gene homolog sequences were obtained and named PCM2, 3, and 4 (respectively annotated GSPATG00034160001, GSPATG00026849001, and GSPATG00036902001 in ParameciumDB). According to the database, PCM1, 2, and 4 are paralogs derived from whole-genome duplications, while PCM3 is not. It must be noted that the sequence of PCM3, reported in the paper, does not exactly match, in its first 90 N-terminal amino acids, to that of GSPATG00026849001. In this protein database, PCM1, 2, and 4 are annotated as harboring a β/γ crystallin domain at their C-terminus, while PCM3 did it at its N-terminus. This domain was already observed in the original paper [121], though ascribed to the C-terminal regions of all four PCMs. In addition, an insulin-like growth factor binding protein, the N-terminal domain, is defined in the protein database. Again, the location of this latter domain varies among PCM; it is either at the N-terminal of PCM1, 2, and 4, or at the C-terminal of PCM3. Therefore, it is like PCM3 was upside down with respect to PCM1, 2, and 4.

### 4.5. Amino Acid Sequences

The amino acid sequence of wild-type CaM from *Paramecium* was obtained using automated Edman degradation combined with mass spectrometry of peptides upon HPLC purification, after digestion with *Staphylococcus aureus* V8 protease [123]. Previously, the only available CaM sequence in ciliates was that of *Tetrahymema* [124].

A linear sequence of 145 amino acids long was raised in wild-type *Paramecium* [123]. Most of the residues were determined by automated Edman degradation, but some other residues were deduced through the total amino acid composition. The most salient feature of the sequenced protein in *Paramecium* was the presence of dimethyl-lysine at position 13, a posttranslational modification that had never been observed before at this position. The most common lysine methylation, trimethyl-lysine at position 115 [124], was also detected. In addition, the wild-type protein was shown to have some changes with respect to vertebrate CaM at the relevant residues, such as Gln at position 79, Asp at position 119, and Val at residue 146. In human CaM, residue 79 is a Thr, residue 119 is a Glu, and residue 146 is a Thr. Despite these differences mapping the α-helical structures that flank the Ca^2+^-binding loops, whose function is unknown, the authors claimed that these residues might not change the structure of the protein and still allow the interactions with target proteins.

We used the published CaM amino acid sequence [123] to find the corresponding sequence in ParameciumDB; as mentioned above, a simple search for “calmodulin” or “CaM” gave too many entries that were not shown. Then, we made use of a pairwise search using the Blast tool, limiting it to *P. tetraurelia* strain d4-2, whose genome was the first to be obtained. This search gave raise to ten hits (not shown), whose alignments showed bit scores ranging from 741 to 307, and E values ranging from 1e^−102^ to 4e^−36^. Strikingly, up to four of these hits were described as “calmodulin”. This finding was contradictory with the fact that CaM was a single gene in *P. tetraurelia* [125]. However, only the sequence that aligned with the best hit, identified as GSPATG00015825001, showed 100% coverage and 100% identity against the query. The other 3 hits (GSPATG00037156001, GSPATG00000755001, and GSPATG00038279001) did not align in its entire sequence with the query.

GSPATG00015825001 is translated into a 149 amino acids protein (considering the initial Met), while GSPATG00037156001, GSPATG00000755001, and GSPATG00038279001 are translated into 146, 152, and 132 amino acids proteins, respectively. Thus, based solely on size criteria, CaM must be a 149 amino acids protein (see Section 2), we considered GSPATG00015825001 as a true or genuine CaM, but we wondered whether the three other sequences could represent CaM paralogs.

To shed light on this issue, we decided to scrutinize the outcome obtained following the orthogroup analysis, with which we inferred the phylogenetic tree shown above (see Section 3). We found sequence GSPATG00015825001 (the genuine CaM) in orthogroup OG0001068 (see Table 1), which also included GSPATP00037156001 (146 amino acids long). Intriguingly, the other *Paramecium* species included in the analysis contained two or more sequences in OG0001068 (see Table 1 and Table 2), except *P. novaurelia*, which showed a single sequence. Likewise, *P. falciparum*, the species used as the outgroup in the phylogenetic tree shown above (see Section 3), had two sequences annotated as CaMs, XP_001348497.1 and XP_001347585.1, of 149 and 242 amino acids long, respectively, clearly showing that only the first one is indeed a genuine CaM.

**Figure 2:**
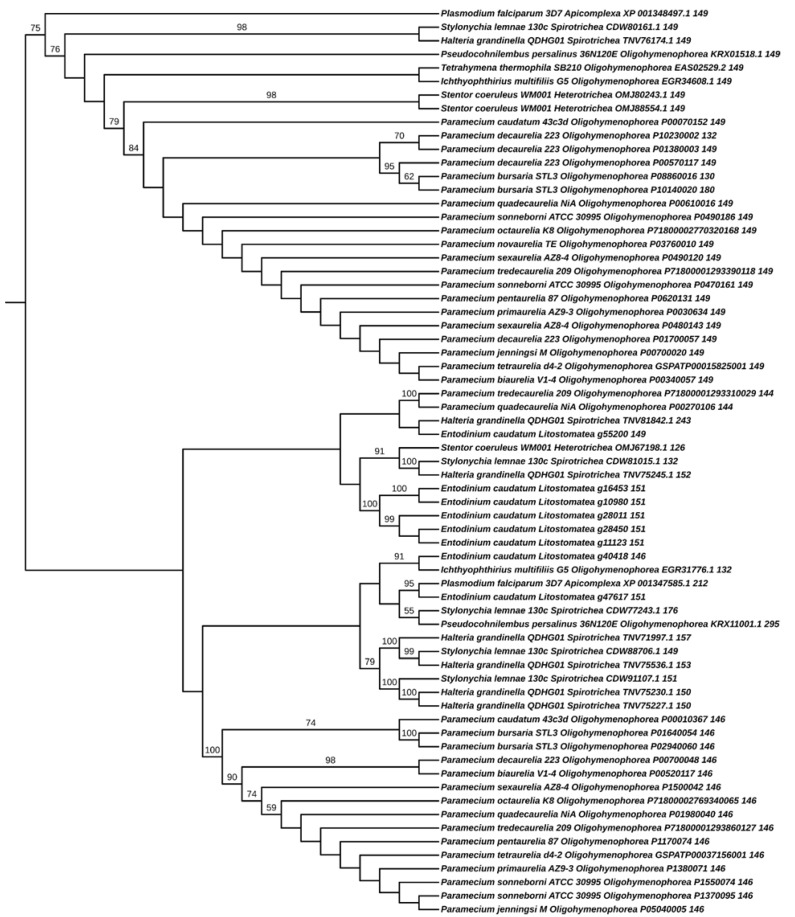
Phylogenetic tree of CaM. Protein sequences from orthogroup OG0001068 were aligned using MAFFT [126]. Alignment file was used with IQ-TREE [127] for tree reconstruction. LG + G4 was the best model selected by IQ-TREE. An Ultrafast Bootstrap approximation with 1000 replicates was used to estimate the node support. However, only bootstrap values higher than 51% are shown. Branch lengths were not considered in order to simplify the tree. The inferred tree was drawn with iTOL (see Figure 1). This tree was rooted so that the two *P. falciparum* sequences were separated from each other, instead of using them as outgroups. For each species, the full name, strain, Class to which each species belongs (except for *P. falciparum*, for which the Phylum was used instead of the Class), database accession number, and amino acids size are shown from left to right, respectively.

Then, we decided to build a new phylogenetic tree, but this time using only sequences found in OG0001068. This tree was rooted in such a way that the two *P. falciparum* sequences were separated from each other, instead of using them as outgroups. As seen in Figure 2, this rooting clustered the sequences into a size-consistent group and a size-heterogenous group. The size-consistent group includes 149 amino acid-long sequences, except three sequences of 132, 130, and 180 amino acids long (the first from *P. decaurelia* and the remaining from *P. bursaria*). In the second cluster (size-heterogenous group), sequences were varied in size, with the included sequences ranging from 126 to 295 amino acids long. In the second group, most of the *Paramecium* sequences were 146 amino acids long and clustered together, except two sequences of 144 amino acids long.

To summarize all these results, we tabulate the sequences into two columns (Table 2): TRUE for all the sequences clustering within the size-consistent group (149 length) and LIKE for all the sequences clustering within the size-heterogenous group (non-149 length). Most ciliates showed 1 or 2 sequences in the TRUE column, except for *P. decaurelia*, showing four sequences in it, and *Entodinium caudatum*, showing no sequence in it. Furthermore, we observed that neither *T. termophila* nor *P. novoaurelia* had sequences in the LIKE column.

**Figure 3:**
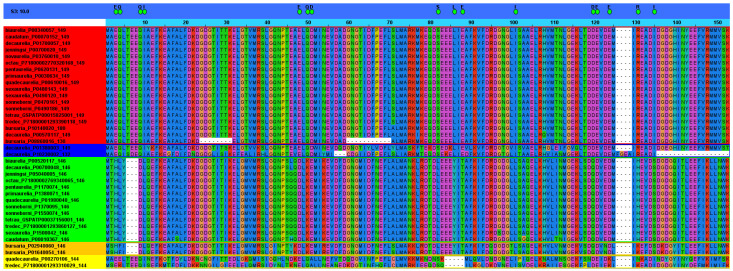
Specificity determining the positions in a multiple alignment of *Paramecium* CaMs. The *Paramecium* subgroup of the protein sequences from orthogroup OG0001068 were aligned using MAFFT [126] and the resulting file uploaded in JDET [129]. Specificity-determining positions were calculated using the S3DET algorithm [129]. The figure shows a partial alignment of the output window. According to this analysis, uploaded CaM sequences were divided in five specificity subgroups, which are highlighted in red, blue, green, orange, and yellow boxes in the leftmost column of the window. This column corresponded to the identification of the proteins, but due to space restrictions, only the species name, accession number, and size of the corresponding proteins were written. The green circles mark the alignment positions that are SDPs allowing discrimination among groups.

We decided to further analyze *Paramecium* sequences from both the TRUE and LIKE groups. All these sequences (130, 132, 144, 146, 149, and 180 amino acids long) were aligned and subdivided into subgroups according to specificity-determining positions (SDP). SDP analyses (for a review, see [128]) allow to discriminate proteins with different functional specificities within a protein family, thus providing a more complete picture of the organization of the family. Fully conserved positions are usually related to functional features common to all the members of the family, while SDP are related to functional specificity. As seen in Figure 3, SDP analysis divide *Paramecium* TRUE and LIKE sequences in four subgroups. The first group contain all the 149 amino acid-long proteins, except for one belonging to *P. decaurelia*, which is grouped differently. Interestingly, the sequences of *P. bursaria* (130 and 180 amino acids long) were grouped within the first group, suggesting that further studies should be performed to uncover the true nature of these proteins. We then wondered whether their “aberrant” size could be due to a wrong prediction of the open reading frames; however, this should be further assessed. Strikingly, two sequences of *P. decaurelia* (132 and 149 amino acids long), which clustered TRUE in the phylogenetic tree, were excluded from the first SDP group.

In summary, according to the data above, most *Paramecium* species (10/14) possess a single, genuine CaM gene. At the most, some species (*P. sexaurelia*, *P. decaurelia*, *P. sonneborni*, and *P. bursaria*) possess two genuine CaM gene copies. Overall, these results suggest that ciliates would not tolerate the presence of more than one genuine CaM gene, and that, in the event of whole-genome duplications, selection would favor the loss of the duplicated copies.

The assumption above contrasts with what is observed in metazoans, where two or more CaM genes coding for an identical CaM protein sequence coexist. For instance, three distinct genes in humans [130] and two in chicken [131,132] have been found. Intriguingly, the amino acid sequences of these multiple, distinct genes are identical. The understanding of the function of each gene is still debated. Plants possess an even higher CaM genetic redundancy, since up to 7 CaM genes coding for distinct CaM protein isoforms are found in the genome of *Arabidopsis* [133].

#### 4.5.1. Mutant Versions

In a way similar to that described in the section above for wild-type CaM, the sequence of CaM from a pantophobiac *Paramecium* mutant, called pantA^1^ (and later renamed as cam^1^, see below), was obtained [134]. However, in this case the protein was purified differently, using a combination of phenyl-Sepharose chromatography, anti-CaM antibody affinity chromatography, and reverse-phase HPLC chromatography. As the wild-type protein, the pantA^1^ CaM was shown to contain dimethyl-lysine at position 13 and trimethyl-lysine at position 115. More importantly, a change in Phe101Ser was found in the pantA^1^ CaM sequence. Noteworthy, position 101 lies in the third Ca^2+^-binding loop and is usually occupied by an amino acid with a hydroxyl-containing side chain, as in *Tetrahymena*, where a Thr residue is found at this position [124]. Nonetheless, the authors claimed that the presence of a bulky side chain, or the absence of a hydroxyl side chain in Phe, resulted in the disruption of the in vivo activity of CaM in this pantophobiac mutant.

The effects of mutations at position 101 were further analyzed by computer modeling, and by producing site-specific mutations at this position and analyzing its activity in vivo and in vitro [92]. Specifically, these point mutations were Ser101Phe (phenylalanine having a nonpolar aromatic ring), Ser101Ala (alanine having a nonpolar methyl group), Ser101Gly (glycine having a hydrogen side chain), and Ser101Tyr (tyrosine having a phenolic hydroxyl group). Computer-aided modeling of SYNCAM [90] and the corresponding Ser101Phe mutant showed no significant differences in structures, since the side chain at residue 101, either a Ser or a Phe, is exposed towards the surface in both of them. As mentioned above (see Section 4.3), SYNCAM is a fully functional CaM that has been synthetically designed [90]. Regarding the restoration of the wild-type phenotype in pantophobiac mutants, SYNCAM calmodulin was able to restore it temporarily, while the Ser101Phe and Ser101Ala mutants did not. From its side, Ser101Gly and Ser101Tyr mutants showed some restorative activity, though one order of magnitude less than CaMs with Ser101. In addition, all the mutated versions at residue 101 activated the chicken gizzard MLCK activity to the same extent, with the Ser101 and Phe101 CaMs being almost identical in kinetic properties as well. Notwithstanding, the molecular mechanism whereby the Ser101Phe mutation alter the locomotion phenotype in *Paramecium* is unknown. The authors of [92] explained this considering three possible scenarios: changes in the Ca^2+^ macroscopic binding constants in CaM, changes in CaM: protein interactions, or a combination of both.

Interestingly, the phenotype of the pantA^1^ mutant (cam^1^ mutation) could be reversed by suppression [135]. Suppressor mutants were obtained by placing pantA^1^ mutants into medium containing increasing amounts of Ba^2+^. Barium is a toxic ion that enter cells through Ca^2+^ channels and, thus, wild-type cells suspended in Ba^2+^ die after some time, but pantA^1^ cells die sooner. Therefore, pantA^1^ cells surviving for longer periods of time in the presence of Ba^2+^ were taken as suppressors for the cam^1^ mutation. Indeed, a suppressor mutant (named cam101), showing both normal activity of the Ca^2+^-dependent K^+^ current and behavioral responses, had an intragenic mutation in cam^1^, according to genetic, electrophysiological, and microinjection data. Nonetheless, this mutation rendered a CaM protein with mobility on acid-urea gels that was different to that of wild-type CaM, and with lesser affinity towards a monoclonal antibody raised against wild-type *Paramecium* CaM [74]. In addition, the cam101 CaM had a PDE-stimulating activity that was comparable in all respects to the wild-type one (see above, Section 4.3), specifically when essaying the activity at a low Ca^2+^ concentration, at which cam^1^ failed to stimulate PDE activity. Finally, it was found that both cam^1^ and cam101 CaMs failed to bind some unidentified CaM-binding proteins that were the target of wild-type CaM. Authors stated that they had a manuscript in preparation about these unidentified CaM-binding proteins, but after searching at NCBI databases we did not find it. Thus, the only available information about these proteins is their molecular mass of 38, 60, and 85 kDa [135].

Alternatively, the sequence of the second pantophobiac mutant, called pantA^2^ (and later renamed as cam^2^, see below) was also raised [91]. In this case, CaM was purified using three consecutive chromatographic steps: phenyl-Sepharose, DEAE-cellulose ion-exchange, and reverse-phase chromatography. The mutation in this case consisted of a point change (Ile136Thr). Residue 136 lies in the fourth Ca^2+^-binding loop, and it is either Val or Ile in most CaMs. Interestingly, dimethyl-lysine at position 13 was also found. Conversely, the data showed different Lys methylation states at position 115, including unmethylated; note that this position is trimethylated in both wild-type and pantA^1^. Interestingly, a cam^2^ recombinant CaM, produced in bacteria, showed to have lower Ca^2+^-binding affinity, as well as to be completely unmethylated at low Ca^2+^ concentration [136]. In addition, data suggested a difference in the conformation of this recombinant cam^2^ that altered the Ca^2+^-depleted structure, probably leading to a lower methylation state at physiological Ca^2+^ concentrations [136]. In spite of this, pantA^2^ CaM was able to stimulate MLCK activity to a level comparable to SYNCAM. Whether this CaM allele was able to restore the locomotion defects was not assessed.

The amino acid sequences of a few other mutant strains were also reported, though in these cases they were either deduced from the nucleotide sequences of different mutant alleles [125] or not provided [55]. The alleles coming from paranoic mutants were named cam^3^ [125], cam^6^, and cam^7^ [55]. As stated above (see Section 3.1), paranoic mutants were characterized by a violent and longer avoiding reaction. The alleles deriving from different Fast-2 mutants were named cam^11^, cam^12^, and cam^13^ [125]. As stated above, the mutant alleles pantA^1^ and pantA^2^ were named cam^1^ and cam^2^, respectively.

The deduced amino acid sequences of cam^1^ and cam^2^ confirmed the mutations previously observed, namely, Ser101Phe in cam^1^ and Ile136Thr in cam^2^. Mutants cam^3^ and cam^11^ showed a single amino acid change with respect to the wild-type, Met145Val and Glu54Lys, respectively. Mutants cam^12^ and cam^13^ showed two amino acid changes with respect to the wild-type, Gly40Glu and Asp50Asn, and Val35Ile and Asp50Asn, respectively. The mutations of cam^1^, cam^2^, and cam^3^ were all located at C-terminal lobe of CaM, while cam^11^, cam^12^, and cam^13^ were all located at N-terminal lobe of CaM. This latter set of mutants eliminated negatively charged residues from the N-terminal lobe, which can be important for the interaction with the positively charged residues of the putative target proteins.

Noteworthy, cam^1^, cam^2^, and cam^3^ mutants showed an overreacting behavior, whereas cam^11^, cam^12^, and cam^13^ mutants were underreactors. These mutants also showed a differentiated response upon cooling; cam^1^ and cam^2^ mutants, like the wild-type cells, increased transiently the frequency of directional changes, while the cam^12^ and cam^13^ mutants showed almost no increase in their frequency [137]. In addition, cam^1^, cam^2^, and cam^3^ mutants were deficient in, or lacked, the outward Ca^2+^-dependent K^+^ current [125,138,139]. Conversely, cam^11^, cam^12^, and cam^13^ were shown to have a deficient inward Ca^2+^-dependent Na^+^ current. At least for the cam^12^ mutant, the current response was restored by adding a phosphodiesterase inhibitor or by injecting hydrolysis-resistant cyclic nucleotides [137].

In a different set of experiments [140], it was shown that injection of antisense oligonucleotides, complementary to CaM mRNA, provoked a decrease in the backward swimming of wild-type cells upon Na^+^ stimulus, which was reversed by the injection of CaM. Injection of these oligonucleotides in cam^1^ mutant cells caused a reduction in their behavioral response, indicating that this mutant is indeed affected in its Ca^2+^-dependent K^+^ channel. Conversely, injection of these oligonucleotides in the cam^11^ mutant caused no change in their behavioral response, indicating that this mutant is indeed affected in its Ca^2+^-dependent Na^+^ channel.

In summary, the outward and inward currents seemed to be differently affected by either the C-terminal or the N-terminal lobe of CaM. Since the C-terminal lobe binds Ca^2+^ with higher affinity than the N-terminal lobe [141,142], it was concluded that the Ca^2+^-dependent K^+^ channel (outward current) might be activated by the half-filled Ca^2+^-CaM, while the Ca^2+^-dependent Na^+^ channel (inward current) might be activated by the completely filled Ca^2+^-CaM.

Heat-treated cytosolic extracts from wild-type and cam^1^, cam^2^, cam^3^, cam^11^, cam^12^, and cam^13^ cells were electrophoresed in denaturing conditions, and probed by immunoblotting with a monoclonal antibody raised against *Paramecium* CaM [77]. This monoclonal antibody [74] detected all mutant CaMs, but it was noticed that proteins from cam^11^, cam^12^, and cam^13^ did not exactly comigrate with the wild-type one [125] Again, each set of mutants shared a common property.

The phenotypes of cam^1^, cam^6^, and cam^7^ mutants were also studied under a different point of view, that of secretion [55]. The main secretory organelles in *Paramecium* are the trichocysts (see Section 3.2), which can be extruded upon different external stimuli. One of these stimuli, picric acid, has no biotic nature, being xenobiotic. Treatment with picric acid kills cells, but also led to trichocyst discharge [56]. Thus, it was assumed that if CaM had been purified from and localized in extruded trichocysts [69,70], then, mutant strains could have had defective secretion of trichocysts. Indeed, it was found that the cam^1^ mutant was unable to secrete trichocysts at 35 °C, cam^7^ was unable to grow at this temperature, while cam^6^ displayed wild-type secretion at this temperature [55]. The incapability of cam^1^ extrusion was due to CaM, since microinjection of the wild-type protein restored this defect. At 35 °C, cam^1^ showed normal trichocysts, but the rosette of the particles as well as the underlying connecting material, both of which would be normally present at the exocytotic sites, were lacking. Moreover, transfer of cam^1^ cells to 22 °C led to the recovery of significant exocytotic capacity. Taken all these results together, the authors concluded [55] that CaM was necessary for the assembly of the structures (rosettes and connecting material) that link the membranes to be fused during exocytosis, but unnecessary for other steps in the exocytotic pathway. In other words, CaM exhibited a morphogenetic effect at the docking sites of trichocysts that promoted their maturation.

### 4.6. Methylation

Post-translational methylation of lysyl residues is very common in the proteomes of living beings. Particularly, CaM is the subject of this post-translational modification, usually at Lys115, a solvent-accessible residue frequently found trimethylated, though the methylation state can change depending on different factors [143]. The enzyme responsible for the methylation activity has been conserved through evolution [144]. As mentioned above, *Paramecium* CaM is methylated at Lys13 and 115 and contains trimethyl-lysine [124] and the responsible enzyme, calmodulin-lysine N-methyltransferase, has been characterized [145]. The enzyme was purified through sequential dialysis and several chromatographic steps, to obtain a 6800-fold purified protein with a 15% yield that was electrophoresed in denaturing conditions, revealing a polypeptide of 37 kDa. In vitro, the enzyme was able to methylate CaM, producing mono-, di-, and trimethyl-lysine at position 115. In addition, Ca^2+^, Mg^2+^, Mn^2+^, and Ni^2+^, but not Zn^2+^, stimulated the activity, while S-adenosylhomocysteine, but not sinefungin and tubercidin, inhibited the activity. Dithiothreitol was required for the N-methyltransferase activity.

Purified CaM from pantA^2^ mutants, which had a unmethylated CaM, was substrate for the N-methyltransferase. However, no other proteins other than CaM in *Paramecium* cytosol seemed to be substrate for this enzyme. As mentioned above, an engineered, and bacterially expressed CaM mutant, bearing the Val136Thr change, mimicking that found in cam^2^ (CaM of pantA^2^ mutant), was not methylated in vitro by the sheep brain methyltransferase in the absence of Ca^2+^ [136].

### 4.7. Crystal Structures

The crystal structure of mammalian CaM, purified from rat testes, was the first CaM structure to be obtained [146] and later refined at a 2.2 Å resolution [147]. A few years later, the structure of recombinant CaM of *Drosophila melanogaster* was obtained at the same resolution too [148], and also further refined at 1.7 Å [149]. To determine the structure of *Paramecium* CaM was of particular interest because it contains 17 differences with respect to mammalian CaM. Most of these differences are conservative and scattered throughout the sequence. Nonetheless, many of the changes, specifically the non-conservative ones, are concentrated at the central helix and the C-terminal lobe of the protein. Thus, the structure of CaM from *Paramecium* was first obtained at a 1.8 Å resolution [150] and then refined at a 1.68 Å resolution [151]. In the latter case, the structure was obtained from a recombinant CaM expressed and purified in *Escherichia coli*. Therefore, this structure was obtained from a protein devoid of any post-translational modification.

**Figure 4:**
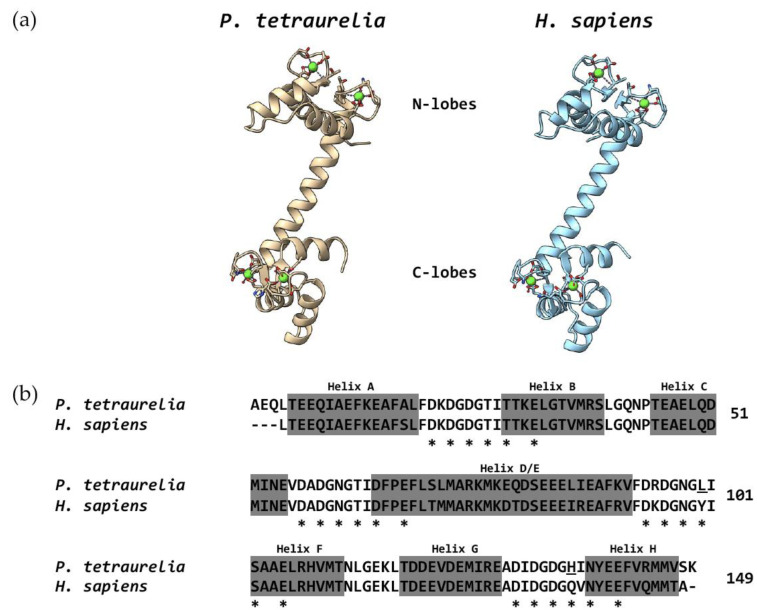
Tertiary structure (**a**) and sequence comparison (**b**) between the CaM of *Paramecium* and humans. (**a**) Sequences were downloaded from the Protein Data Bank under the accession numbers 1OSA (*P. tetraurelia*) and 1CLL (*Homo sapiens*). Drawings using these sequences were made using Chimera X [152]. Green spheres represent Ca^2+^ ions. (**b**) The amino acid sequences in (**a**) were aligned using Chimera X. Note that these sequences are shorter than 149 amino acids since the crystals were obtained using non-full-length CaMs. Dashes indicate that the crystal structure lacked the amino acid at these positions. Stars indicate the amino acids that coordinate Ca^2+^ ions in humans according to [15]. Underlined amino acids mark those ones that are different in *Paramecium* at the Ca^2+^-coordinating sites. Grey-shadowed amino acids correspond to helices (see text). Numbers at the right indicate the amino acid position in the sequence of *Paramecium*.

At the lowest refinement, *Paramecium* CaM was a molecule of 65 × 30 × 30 Å in size. Each globular Ca^2+^-binding domain was 20 × 20 × 20 Å in size [150]. Overall, the structure of the *Paramecium* CaM was similar to that of the mammalian CaM (see Figure 4a for visual comparison), namely, the protein folded to form a dumbbell-shaped molecule, having seven α-helices including the central helix, named D/E helix, that connect the two terminal lobes [151]. The position of these helices was (see Figure 4b): A helix (residues 6–19), B helix (residues 29–39), C helix (residues 45–55), D/E helix (residues 65–92), F helix (residues 102–111), G helix (residues 118–128), and H helix (residues 138–147). In the EF-hand motifs, the four Ca^2+^-binding loops, each 12 residues long and binding one Ca^2+^ ion, were site I (residues 20–31), site II (residues 56–67), site III (residues 93–104), and site IV (residues 129–140).

The most similar sites were I and III, while the least similar were sites I and IV [151]. It was observed, comparing the conformation of the different sites, that there were some apparent conformational differences between site IV and the other sites. The EF-hand motifs were seen to be further stabilized by mini β-sheets, formed between the eighth residues of adjacent loops [151].

*Paramecium* CaM binds four Ca^2+^ ions, all of which were seven coordinated in the loops, with the average coordination distances in the range of 2.30–2.39 Å. The coordination involves the following elements: the side-chain carboxylate O atoms of the first, the third and the fifth residues, the backbone carbonyl O atom of the seventh residue, both the carboxylate O atoms of the twelfth residue, and a water molecule [151].

The N- and C-terminal lobes contained 28 residues that were identical, 26 residues that were conservatively changed, and 9 residues that were different. In spite of this, the side chains of the nine residues that were different had quite similar intra- and intermolecular interactions. Apparently, the C-terminal lobe was slightly deviating from that of other CaMs. In addition, the central helix had eight backbone carbonyl O atoms that were forming hydrogen bonds to water molecules, and it did not present a marked bending.

When comparing the wild-type (post-translationally modified) CaM with the recombinant (non-modified) CaM, differences were seen at Lys13 and Lys115, where the residues in the wild type were di- and tri-methylated, respectively. Though the backbone atoms of these residues have no essential differences, the side-chain conformations were substantially different in both structures [151]. In the wild-type structure, these residues were bent towards the interior of the molecule, while in the recombinant structure the same residues were stretched out into the solvent.

To our knowledge, the crystal structures of neither mutant CaM of *Paramecium* has been made available yet. However, some other analyses, showing interesting clues about the functioning of CaM, were done. As mentioned above, the mutations localized at the N-domain of *Paramecium* CaM (mutants cam^11^, cam^12^, and cam^13^) decreased the Ca^2+^-dependent Na^+^ current, while mutations localized at the C-domain (mutants cam^1^, cam^2^, and cam^3^) decreased the Ca^2+^-dependent K^+^ current. Interestingly, the mutations at the N-domain grouped in Ca^2+^-binding sites I and II, while mutations at the C-domain grouped in Ca^2+^-binding sites III and IV. However, it was shown [153,154] that all mutant CaMs bound Ca^2+^, adopting a Ca^2+^-saturated conformation similar to that of wild-type CaM, as evidenced through several data sets. The Ca^2+^ binding properties of sites III and IV were severely affected in the C-terminal mutants (requiring more free Ca^2+^ to reach saturation), while they did not in the N-terminal mutants. Consequently, it was concluded [153] that Ca^2+^-binding affinities of sites in the C-domain were not affected by mutations in the N-domain. In addition, most of the mutant CaMs adopted conformations, in both the apo and Ca^2+^-saturated states, similar to those of the wild type. However, due to the decreases in Ca^2+^ affinity, some CaM mutants might never reach the required end state at physiological Ca^2+^ levels, hence the difference in the behavior of the mutants in affecting a channel type or the other. Thus, the idea that the N- and the C-terminal domains were not equivalent in activating the target proteins was put forward [153].

### 4.8. Nucleotide Sequences

The nucleotide sequence of the wild-type CaM and mutant alleles (cam^1^, cam^2^, cam^3^, cam^11^, cam^12^, and cam^13^) were obtained [125] by means of cloning the PCR products, which were amplified using degenerated primers. The cloned sequence of the wild type consisted of 445 bp in the coding region, plus 135 and 241 bp, respectively, for the 5′ and 3′ flanking regions. The deduced amino acid sequence matched the sequence obtained by protein sequencing [123] and added information about the order of the three first amino acids. The sequences of mutant alleles were similar to the wild-type one, except in the specific places where the mutation(s) was(were) accumulated (see Section 4.5.1 for the changes these mutations caused in the amino acid sequences).

The coding sequence of wild-type CaM was not interrupted by any introns; it terminated with the TGA codon, and contained five in-frame TAA codons, coding for Gln. These codons represented one of the well-known codon deviations found in *Paramecium* (for a review, see [155]). The coding sequence was 65% AT-rich, while the 5′ and 3′ flanking regions were AT-richer, respectively, 81% and 83%. A putative TATA box was located 92 bp upstream from the initiation codon, while putative polyadenylations signals (AATAAA and ATTAAA) and termination signals (TTTTT) were found in the 3′ flanking region. The *Paramecium* CaM gene was successfully expressed in *E. coli* after changing its four TAA codons [156], which code for Gln in *Paramecium* [155], to CAA (one of the canonical Gln codons).

The CaM gene was used as a probe to determine copy number in the macronuclear genome and to identify the mRNAs species. Results showed that there was a single CaM gene in the macronucleus that produce a single polyA-containing mRNA species. A single CaM gene is usually found in non-metazoans, such as *Tetrahymena* [157], yeasts [158,159], *Plasmodium* [160], and green algae [161], but not in *Trypanosoma cruzi* and *T. brucei*, respectively having eight and three tandemly repeated CaM genes either in one or two different loci [162,163].

As described in Section 4.5, the gene codifying for CaM in *P. tetraurelia* was annotated as GSPATG00015825001 in ParameciumDB. According to this database, the gene has no paralog from whole-genome duplications, as we have also evidenced through the orthogroup and SDP analysis. ParameciumDB also offers some expression profiles for this gene, both at the mRNA and protein levels. On the one hand, transcriptomic data show that the gene is downregulated at the beginning of meiosis, compared to vegetative cells. Finally, the gene raises its expression in a later stage of meiosis, when the macronuclear anlage is visible. On the other hand, proteomic data show that CaM is detected in cilia but not in the ciliary membrane or the infracilliary lattice. These data agree, although only in part, with what had been previously reported, since, as stated above (see Section 4.1), CaM was successfully localized in cilia and the infracilliary lattice.

Using the data obtained after the orthogroup analysis, we decided to examine synteny in the different *Paramecium* species used in this analysis. Thus, we searched for the identities of the genes that are located immediately upstream and downstream of TRUE CaMs (see above, Section 4.5). Then, we assigned the identified genes to their corresponding orthogroup and finally tabulated these data. As seen in Table 3, most *Paramecium* species maintain a certain pattern, both upstream and downstream of CaM, of what we call “orthosynteny”. Thus, the more representative orthogroups upstream of CaM are OG0000201 (annotated in *P. tetraurelia* as starch-binding domain/profilin/carbohydrate-binding module family 20), OG0007300 (annotated in *P. tetraurelia* as ribosome maturation factor RimP/coiled coil domain), and OG0001678 (annotated in *P. tetraurelia* as ATP-NAD kinase C-terminal domain-ATP/NAD kinase N-terminal domain), respectively at −1, −2, and −3 positions. Likewise, the more representative orthogroups downstream of CaM are OG0013227 (annotated in *P. tetraurelia* as protein kinase domain) and OG0001289 (annotated in *P. tetraurelia* as Rer1 family), respectively, the at +1 and +2 positions.

## Figures and Tables

**Table 1:** Listing of sequences in orthogroup OG0001068. The sequences listed here are identified as CaMs according to their presence within the orthogroup of *P. tetraurelia* CaM (GSPATG00015825001). The left column indicates the species to which the sequences shown in the right column belong. Along with the species name, the Class taxonomic rank is included, except in the one marked with an asterisk, which corresponds to Phylum. The sequences are represented by their accession numbers in the corresponding database (see text in Figure 1).

Species, Class	Sequence IDs
*Entodinium caudatum*, Litostomatea	g10980, g11123, g16453, g28011, g28450, g40418, g47617, g55200
*Halteria grandinella* QDHG01, Spirotrichea	TNV71997.1, TNV75227.1, TNV75230.1, TNV75245.1, TNV75536.1, TNV76174.1, TNV81842.1
*Ichthyophthirius multifiliis* G5, Oligohymenophorea	EGR31776.1, EGR34608.1
*Paramecium biaurelia* V1-4, Oligohymenophorea	P00340057, P00520117
*Paramecium bursaria* STL3, Oligohymenophorea	P01640054, P02940060, P08860016, P10140020
*Paramecium caudatum* 43c3d, Oligohymenophorea	P00010367, P00070152
*Paramecium decaurelia* 223, Oligohymenophorea	P00570117, P00700048, P01380003, P01700057, P10230002
*Paramecium jenningsi* M, Oligohymenophorea	P00700020, P05040005
*Paramecium novaurelia* TE, Oligohymenophorea	P03760010
*Paramecium octaurelia* K8, Oligohymenophorea	P71800002769340065, P71800002770320168
*Paramecium pentaurelia* 87, Oligohymenophorea	P0620131, P1170074
*Paramecium primaurelia* AZ9-3, Oligohymenophorea	P0030634, P1380071
*Paramecium quadecaurelia* NiA, Oligohymenophorea	P00270106, P00610016, P01980040
*Paramecium sexaurelia* AZ8-4, Oligohymenophorea	P0480143, P0490120, P1500042
*Paramecium sonneborni* ATCC30995, Oligohymenophorea	P0470161, P0490186, P1370095, P1550074
Paramecium tetraurelia d4-2, Oligohymenophorea	GSPATP00015825001, GSPATP00037156001
*Paramecium tredecaurelia* 209, Oligohymenophorea	P71800001293310029, P71800001293390118, P71800001293860127
*Pseudocohnilembus persalinus* 36N120E, Oligohymenophorea	KRX01518.1, KRX11001.1
*Stentor coeruleus* WM001, Heterotrichea	OMJ67198.1, OMJ80243.1, OMJ88554.1
*Stylonychia lemnae* 130c, Spirotrichea	CDW77243.1, CDW80161.1, CDW81015.1, CDW88706.1, CDW91107.1
*Tetrahymena thermophila* SB210, Oligohymenophorea	EAS02529.2
*Plasmodium falciparum* 3D7, Apicomplexa *	XP_001347585.1, XP_001348497.1

* The taxon is the Phylum.

**Table 2:** Number of sequences in orthogroup OG0001068. The sequences listed in Table 1 were grouped according to the clusters observed in the phylogenetic tree in Figure 2. TRUE sequences are those that cluster along with *P. tetraurelia* CaM (GSPATG00015825001). LIKE sequences are the sequences that cluster apart from GSPATG00015825001. The leftmost column indicates the species to which the number of sequences shown in the next columns belong. Along with the species name, the Class taxonomic rank is included, except in the one marked with an asterisk, which corresponds to Phylum.

Species, Class	TRUE	LIKE	TOTAL
*Entodinium caudatum*, Litostomatea	0	8	8
*Halteria grandinella* QDHG01, Spirotrichea	1	6	7
*Ichthyophthirius multifiliis* G5, Oligohymenophorea	1	1	2
*Paramecium biaurelia* V1-4, Oligohymenophorea	1	1	2
*Paramecium bursaria* STL3, Oligohymenophorea	2	2	4
*Paramecium caudatum* 43c3d, Oligohymenophorea	1	1	2
*Paramecium decaurelia* 223, Oligohymenophorea	4	1	5
*Paramecium jenningsi* M, Oligohymenophorea	1	1	2
*Paramecium novaurelia* TE, Oligohymenophorea	1	0	1
*Paramecium octaurelia* K8, Oligohymenophorea	1	1	2
*Paramecium pentaurelia* 87, Oligohymenophorea	1	1	2
*Paramecium primaurelia* AZ9-3, Oligohymenophorea	1	1	2
*Paramecium quadecaurelia* NiA, Oligohymenophorea	1	2	3
*Paramecium sexaurelia* AZ8-4, Oligohymenophorea	2	1	3
*Paramecium sonneborni* ATCC30995, Oligohymenophorea	2	2	4
*Paramecium tetraurelia* d4-2, Oligohymenophorea	1	1	2
*Paramecium tredecaurelia* 209, Oligohymenophorea	1	2	3
*Pseudocohnilembus persalinus* 36N120E, Oligohymenophorea	1	1	2
*Stentor coeruleus* WM001, Heterotrichea	2	1	3
*Stylonychia lemnae* 130c, Spirotrichea	1	4	5
*Tetrahymena thermophila* SB210, Oligohymenophorea	1	0	1
*Plasmodium falciparum* 3D7, Apicomplexa *	1	1	2

* The taxon is the Phylum.

**Table 3:** Synteny around CaMs in *Paramecium* species. The sequences classified as TRUE in Table 2 were searched for in ParameciumDB and the corresponding upstream and downstream genes localized and assigned to its orthogroup. The column CALMODULIN indicates the accession number of the corresponding CaMs in the different species. To help with visualization, some orthogroup are marked by a different color. Numerals marked with a minus indicate upstream genes, while the other numerals indicate downstream genes.

Species	−4	−3	−2	−1	CALMODULIN	1	2	3
*P. biaurelia*	OG0003656	OG0001678	OG0007300	OG0000201	P00340057	OG0013227	OG0001289	OG0000584
*P. bursaria*	OG0000460	OG0034217	OG0001284	OG0022984	P08860016	OG0004706	OG0034218	OG0010485
*P. bursaria*	OG0011926	OG0034217	OG0001284	OG0022984	P10140020	OG0004706	OG0034218	OG0010485
*P. caudatum*	OG0008990	OG0004005	OG0035853	OG0035853	P00070152	OG0026817	OG0005842	OG0000048
*P. decaurelia*	OG0005051	OG0005051	OG0006772	OG0000494	P00570117		OG0017080	OG0000016
*P. decaurelia*	OG0002384	OG0018936	OG0015720		P01380003	OG0000209		OG0008678
*P. decaurelia*	OG0003656	OG0001678	OG0007300	OG0000201	P01700057	OG0013227	OG0001289	OG0000584
*P. decaurelia*	OG0003883	OG0012438			P10230002	OG0013227	OG0021818	OG0000099
*P. jenningsi*		OG0001678	OG0007300		P00700020			OG0001289
*P. novaurelia*	OG0003656	OG0001678	OG0007300	OG0000201	P03760010	OG0013227	OG0001289	OG0000584
*P. octaurelia*	OG0003656	OG0001678	OG0007300	OG0000201	P71800002770320168	OG0013227	OG0001289	
*P. pentaurelia*	OG0003656	OG0001678	OG0007300	OG0000201	P0620131	OG0013227	OG0001289	OG0000584
*P. primaurelia*	OG0003656	OG0001678	OG0007300	OG0000201	P0030634	OG0013227	OG0001289	OG0000584
*P. quadecaurelia*	OG0003656	OG0001678		OG0000201	P00610016	OG0013227	OG0001289	
*P. sexaurelia*	OG0003656	OG0001678	OG0007300	OG0000201	P0480143		OG0000584	OG0000014
*P. sexaurelia*	OG0014690	OG0010650	OG0011665	OG0003706	P0490120	OG0013227	OG0001289	OG0000014
*P. sonneborni*	OG0003656	OG0001678	OG0007300		P0470161	OG0001289	OG0000584	OG0000014
*P. sonneborni*	OG0003656	OG0001678	OG0007300	OG0000201	P0490186	OG0013227	OG0001289	OG0013227
*P. tetraurelia*	OG0003656	OG0001678	OG0007300	OG0000201	GSPATP00015825001	OG0013227	OG0001289	OG0037287
*P. tredecaurelia*	OG0003656	OG0001678	OG0007300	OG0000201	P71800001293390118	OG0013227	OG0001289	OG0000584

## Data Availability

Not applicable.
